# Complement evasion by *Bordetella pertussis*: implications for improving current vaccines

**DOI:** 10.1007/s00109-015-1259-1

**Published:** 2015-02-18

**Authors:** Ilse Jongerius, Tim J. Schuijt, Frits R. Mooi, Elena Pinelli

**Affiliations:** 1 Centre for Infectious Disease Control, National Institute for Public Health and the Environment, Antonie van Leeuwenhoeklaan 9, P.O. Box 1, 3720 BA Bilthoven, The Netherlands; 2 Present Address: Department of Medical Microbiology, University Medical Center Utrecht, Heidelberglaan 100, 3584 CX Utrecht, The Netherlands; 3 Present Address: Department of Clinical Chemistry, Hematology and Immunology, Diakonessenhuis, Bosboomstraat 1, 3582 KE Utrecht, The Netherlands

**Keywords:** Complement, *Bordetella pertussis*, Innate immunity, Evasion, Vaccine

## Abstract

*Bordetella pertussis* causes whooping cough or pertussis, a highly contagious disease of the respiratory tract. Despite high vaccination coverage, reported cases of pertussis are rising worldwide and it has become clear that the current vaccines must be improved. In addition to the well-known protective role of antibodies and T cells during *B. pertussis* infection, innate immune responses such as the complement system play an essential role in *B. pertussis* killing. In order to evade this complement activation and colonize the human host, *B. pertussis* expresses several molecules that inhibit complement activation. Interestingly, one of the known complement evasion proteins, autotransporter Vag8, is highly expressed in the recently emerged *B. pertussis* isolates. Here, we describe the current knowledge on how *B. pertussis* evades complement-mediated killing. In addition, we compare this to complement evasion strategies used by other bacterial species. Finally, we discuss the consequences of complement evasion by *B. pertussis* on adaptive immunity and how identification of the bacterial molecules and the mechanisms involved in complement evasion might help improve pertussis vaccines.

## Introduction

**Fig. 1:**
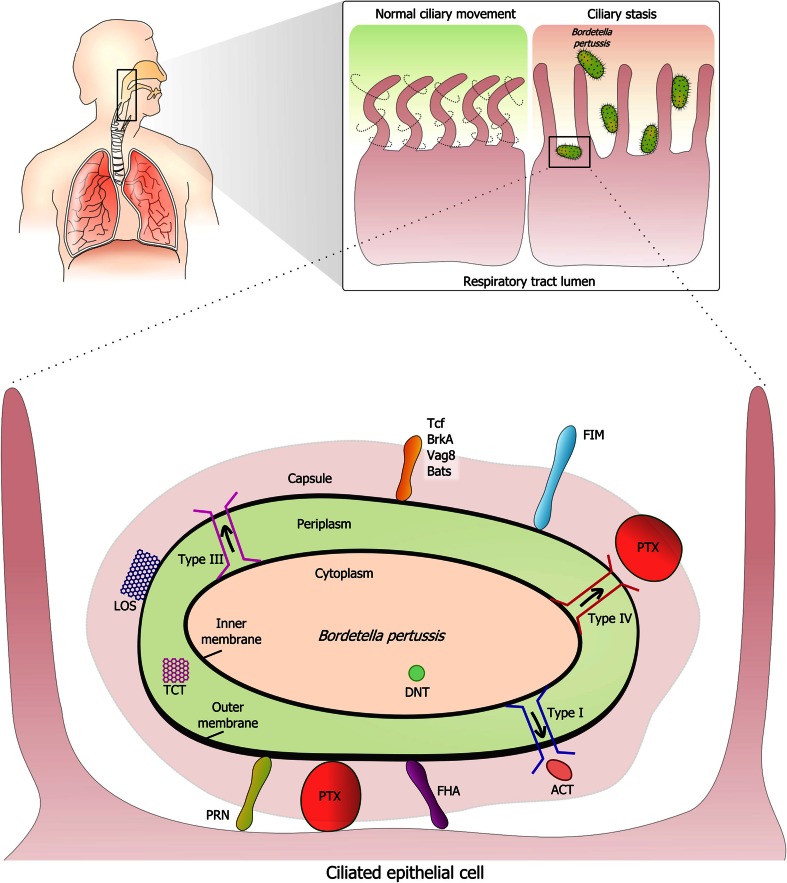
Interaction of *B. pertussis* with mucosal surfaces. The Gram-negative bacterium *B. pertussis* interacts with ciliated epithelium in the respiratory tract. Cilia are found in the trachea, bronchi, and bronchioles and move continuously to keep the airway free of mucus-trapped microorganisms and dust. A number of *B. pertussis* proteins have been implicated in adherence to host receptors, including pertactin (Prn), cell-bound pertussis toxin (Ptx), filamentous hemagglutinin (FHA), fimbriae (Fim), tracheal colonization factor A (TcfA), *Bordetella* resistance to killing protein A (BrkA), the autotransporter Vag8, and other *Bordetella* autotransporters (Bats). Ptx and adenylate cyclase toxin (ACT) are toxic for host cells including phagocytes. *Bordetella* dermonecrotic toxin (DNT) induces vasoconstriction in vitro. *B. pertussis* contains lipooligosaccharide (LOS, or endotoxin) in its outer membrane. Type I, III, and IV secretion systems are indicated in *red*, *blue*, and *pink*, respectively. Ciliostasis is induced by tracheal cytotoxin (TCT) and may induce bouts of intense coughing in whooping cough patients in order to remove accumulated mucus

The Gram-negative bacterium *Bordetella pertussis* causes pertussis or whooping cough, a highly contagious disease of the respiratory tract of humans. *B. pertussis* is primarily transmitted via direct contact or inhalation of airborne droplets expelled by infected individuals while coughing [1, 2]. Upon infection, the bacteria attach to ciliated epithelium of the upper respiratory tract where they multiply and express various virulence factors that favor colonization (Fig. 1) [2, 3]. These virulence factors include, e.g., membrane-bound molecules involved in adherence to the ciliated cells, secreted toxins, and proteins that affect complement-mediated killing. Frequently associated complications of pertussis are pneumonia, otitis media, seizures, and (brain) hemorrhages [4]. Pertussis was a leading cause of infant death before the introduction of the whole-cell pertussis (wP) vaccines in the 1950s. Due to side effects of the wP vaccine, acellular pertussis (aP) vaccines were introduced in the late 1990s [5]. Despite high vaccination coverage, reported cases of pertussis have been increasing over the past three decades [6, 7]. Possible explanations for the re-emergence of pertussis are the limited duration (waning) of aP vaccine-induced immunity and pathogen adaptation. Other influencing factors may be the increased awareness of disease and better diagnostic tools for detection of pertussis [6, 8, 9].

To establish colonization and infection, pathogens have developed various mechanisms to evade host immune responses including the complement system [10]. *B. pertussis* is not an exception to this phenomenon. Here, we review what is known about the interactions between *B. pertussis* and the complement system and how this pathogen evades complement-mediated killing. In addition, we discuss how identification of the molecules and the mechanisms involved in complement evasion might help to improve the current *B. pertussis* vaccines.

## The role of complement in the host’s defense against *B. pertussis*

**Fig. 2:**
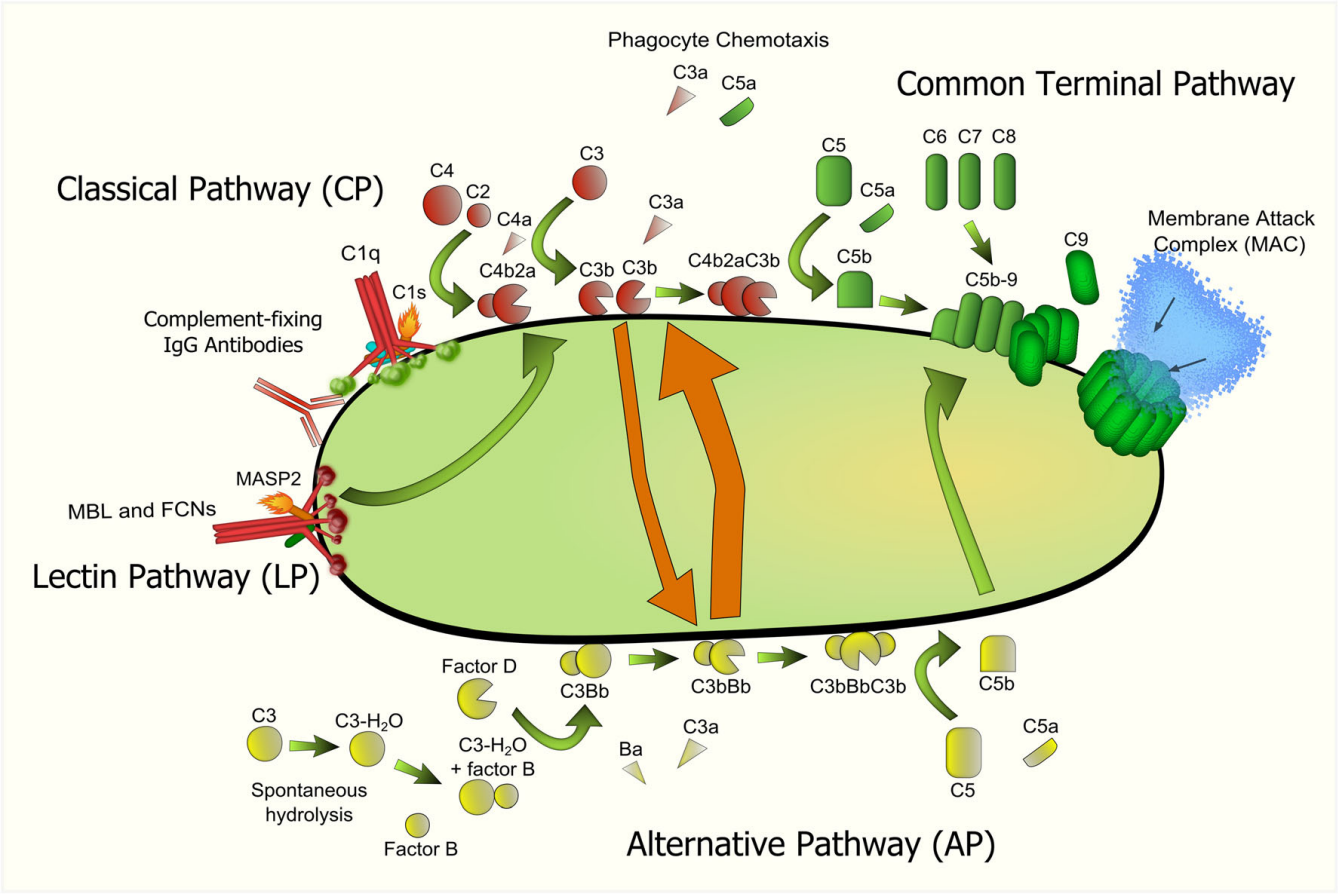
Complement activation pathways. Complement activation is mainly initiated via the CP and LP of complement. Initiation of complement activation on the bacterial surface occurs via either C1q of the CP or via mannose-binding lectin (MBL) or ficolins (FCNs) of the LP and is indicated in *red*. Complement-fixing IgG, bound to the surface of *B. pertussis*, activates the CP of complement. C1q, in complex with proteases C1r and C1s binds to the bacterial surface and activates complement C2 and C4. Similarly, MBL and/or FCNs are in complex with serine protease MASP-1/-2/-3 (mannose-binding lectin-associated serine protease-1/-2/-3) and binding the pathogen surface leads to autoactivation of MASP-2, allowing cleavage of C2 and C4. C3 convertases can either cleave additional C3 into C3b, or bind C3b, producing the C5 convertase (C4bC2aC3b). C5 convertases cleave C5 which in turn leads to the formation of the terminal pathway (indicated in *green*) which produces the membrane attack complex (MAC). The MAC is formed through the terminal assembly of complement components C5b through C9 and results in cell lysis and death of Gram-negative bacteria. The AP (indicated in *yellow*) involves the continuous spontaneous hydrolysis of C3 into C3-HO, which binds to factor B, producing Bb and Ba through the action of factor D. Properdin binds and stabilizes the alternative C3 convertase C3bBb. The latter can either cleave more C3 or forms the C5 convertase by incorporation of another C3b molecule, producing the C5 convertase (C3bBbC3b). The AP can be activated spontaneously and it can also amplify the other pathways (as indicated with the *orange arrows*). In addition to MAC formation, activation of the complement cascade results in leukocyte chemotaxis and opsonization of the invading pathogen, leading to enhanced phagocytosis

The human complement system serves as the first line of defense against microorganisms. The complement cascade can be activated via three different pathways: the classical (CP), lectin (LP), and/or the alternative pathway (AP) (Fig. 2). Activation of any of the three pathways results in cleavage of C3 by C3 convertases which in turn leads to opsonization of bacteria with C3b. C3b deposition greatly enhances bacterial uptake by phagocytic cells and can form a bridge between the innate and adaptive immunity [11, 12]. In addition, C3b deposition on the bacterial surface leads to formation of C5 convertases which cleave C5 into the strong chemo-attractant C5a and C5b, which in turn initiates the formation of the membrane attack complex (MAC) that can lyse Gram-negative bacteria [13]. To prevent damage to human cells, the complement system is strictly regulated and several complement regulatory proteins are described that can inhibit the CP, LP, or AP [14]. Complement is not only present in the blood, but also on healthy human mucosal surfaces of the upper respiratory tract and the lungs. Moreover, almost all cells in the human body can produce complement proteins (reviewed in [15]). Therefore, complement can interfere with successful colonization and persistence of bacteria in the upper respiratory tract and the lung [16–18].

In addition to direct killing of invading microbes, complement activation is also involved in other biological processes of the human body [14] including the development and modulation of adaptive immune responses. Although it has long been acknowledged that complement plays a role in regulating B cell immunity [19], it has only recently become clear that complement is also involved in inducing and directing T cell responses [20]. This activation can occur through direct modulation of the T cells themselves, or indirect activation through alteration of mainly antigen presenting cells [21–23]. Furthermore, it has been shown that complement receptor-mediated signaling can act in synergy with different innate receptors such as Toll-like receptors, which promotes for example, Th17 differentiation which has been reported to be important for protection against *B. pertussis* infections [24, 25]. Inhibition of complement activation by *B. pertussis* can therefore also have consequences for the induction of the T cells required for protection against this pathogen.

## Complement evasion strategies of *B. pertussis*

**Fig. 3:**
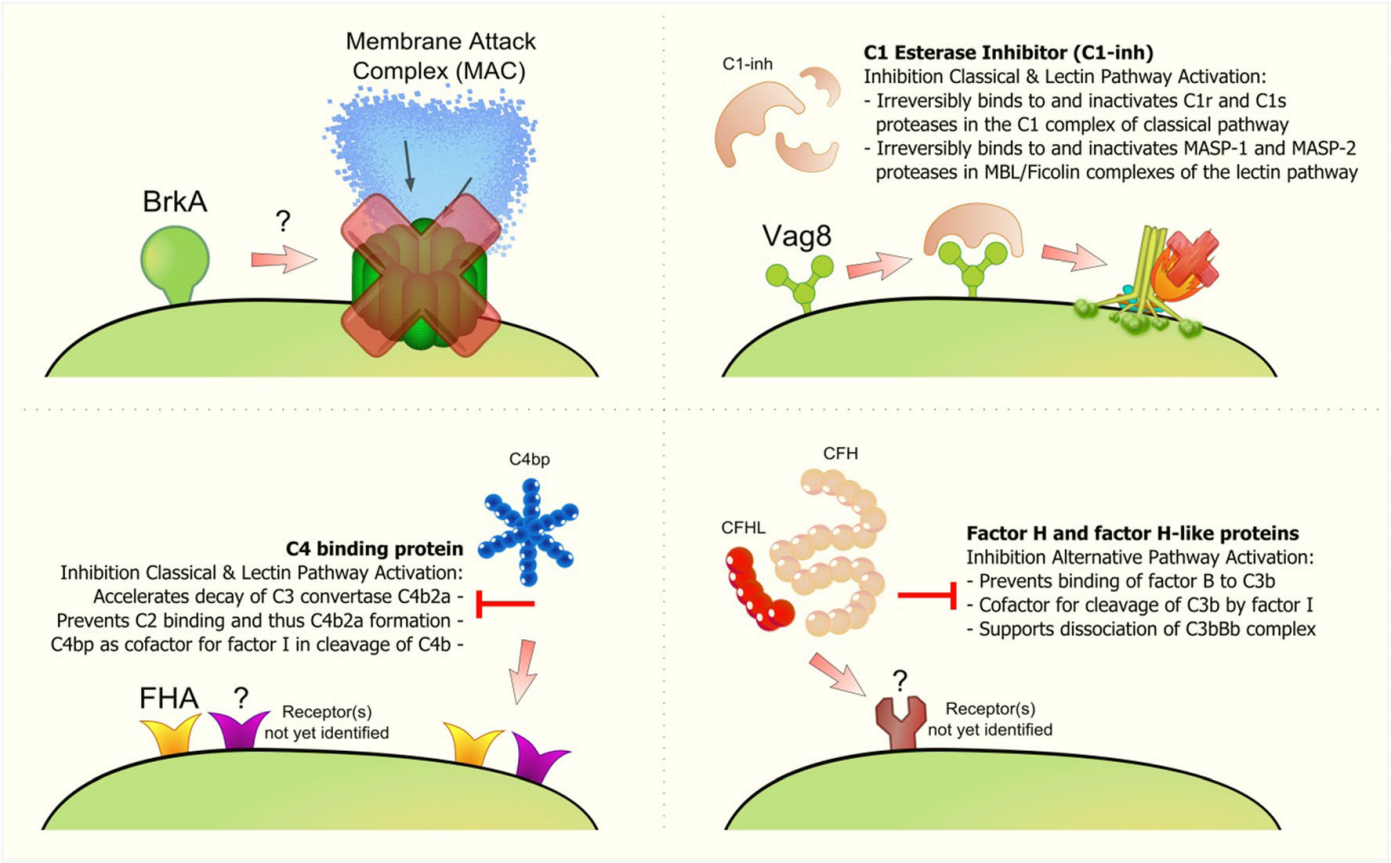
Complement resistance mechanisms of *B. pertussis. B. pertussis* has evolved several strategies to evade complement activation. **a** BrkA, an autotransporter of *B. pertussis*, has been shown to be involved in complement evasion. The exact mechanism of how BrkA inhibits complement activation remains unknown. **b** *B. pertussis* binds C1-inh to the bacterial surface which increased resistance to complement-mediated killing. The Vag8 protein of *B. pertussis* was identified as the C1-inh binding factor. **c** *B. pertussis* binds C4BP via its surface protein FHA and possibly via one or more other receptors. Strains deficient in FHA were still able to bind C4BP, although strongly reduced. **d** *B. pertussis* isolates recruit host complement regulators that are part of the fH family such as complement CFH, factor H-like 1 (CFHL), and factor H-related (CFHR) proteins by expressing one or more receptors that have not yet been identified

In order to infect and survive in the human host, pathogens employ a broad range of strategies to escape recognition and killing by various immune mechanisms, including the complement system [10], and *B. pertussis* is no exception to this phenomenon. Although research on the interaction with *B. pertussis* and the innate immune system is limited, it is known that in order to escape the complement system, this bacterium expresses several complement evasion molecules. Unlike *Bordetella parapertussis* and *Bordetella bronchiseptica*, which can cause infectious bronchitis in humans [7], *B. pertussis* does not expresses lipopolysaccharide containing O-antigen. Murine infection models show that lipopolysaccharide containing O-antigen facilitates colonization of the respiratory tract of mice and also plays an important role in the protection against complement-mediated killing since it prevents C3b deposition on the bacteria surface [26, 27]. In addition to O-antigen, other surface polysaccharides have also been shown to provide complement resistance [28, 29]. Like other bacterial pathogens, *B. pertussis* expresses a polysaccharide (Bps) which belongs to a large family of β-(1-6)-linked polymeric-N-acetylglucosamine (GlcNAc) polysaccharides. Bps polysaccharide was shown to be essential for early colonization of the respiratory tract of mice by *B. pertussis* [30, 31]. Recent studies show that Bps mutant strains are more sensitive to complement-mediated killing compared to the *B. pertussis* wild-type strain [32, 31]. As mice are not the natural reservoir of *B. pertussis* and its pathology of infection is different from humans, further studies are needed to establish the effect of surface polysaccharides during infections in humans. In addition to surface polysaccharides, *B. pertussis* expresses several other proteins involved in complement evasion (Fig. 3) which are described in detail below.

The 103-kDa autotransporter, *Bordetella* resistance to killing A (BrkA) protein of *B. pertussis* promotes attachment to human cells and has also been shown to be involved in complement evasion [33] (Fig. 3a). Studies using a BrkA mutant and a BrkA overexpressing *B. pertussis* strain demonstrated that BrkA reduces C4 and C3 deposition on the bacterial surface and subsequently, the formation of the MAC complex [34]. Since C1q deposition was not altered by BrkA, the authors conclude that BrkA either promotes degradation of C4b on the bacterial surface or inhibits C4 activation [34]. The authors did not investigate a potential direct effect of BrkA on C1, C2, and/or C4. Direct binding of BrkA to those proteins could result in conformational changes of these proteins, thereby preventing their activation. Another possibility is inactivation of C1, C2, and/or C4 by BrkA via proteolytic cleavage. Furthermore, all studies were performed with whole bacteria rather than recombinant BrkA so it cannot be excluded that BrkA is indirectly involved in complement inhibition rather than directly. Therefore, the precise mechanism by which BrkA inhibits complement activation remains to be fully elucidated. Recent studies also identified another autotransporter protein, *Bordetella* autotransporter protein-C (BapC) that is involved in serum resistance. Although it was shown that a BapC mutant of *B. pertussis* is more susceptible to serum killing, the mechanism of action remains unidentified [35].

A frequently used mechanism by pathogens to evade complement-mediated killing is the acquisition of host complement regulators on their surfaces [10]. The host complement regulator C1 esterase inhibitor (C1-inh) belongs to the superfamily of serine protease inhibitors. C1-inh binds irreversibly to and inactivates both C1r and C1s proteases of the CP and mannan-binding lectin-associated serine protease (MASP)-1 and MASP-2 of the LP [36]. *B. pertussis*, but not the related *B. bronchiseptica*, *B. parapertussis*, *Bordetella holmesii*, or *Bordetella avium*, recruits C1-inh to its bacterial surface to inhibit complement activity [37]. The binding of C1-inh to the bacterial surface is dependent on the expression of genes that are under control of the *Bordetella* master virulence regulatory locus (bvgAS) [37]. The BvgAS proteins form part of a two-component sensory transduction system which is regulated by environmental signals. Growth in the presence of sulfate, nicotinic acid, or low temperature results in lack of expression of the *bvg*-activated genes and results in an avirulent phenotype of this bacterium [38]. Using different growth conditions, the authors showed that C1-inh binding occurred only during the virulent phase. Recently, the passenger domain of the autotransporter Vag8 was identified as the C1-inh binding factor of *B. pertussis*. Importantly, Vag8 expression correlates with serum resistance [39] (Fig. 3b). Interestingly, the *B. pertussis ptxP3* (or P3) lineage which has recently expanded globally, produces higher amounts of Vag8 than the strains it replaced [40]. In addition to *B. pertussis*, only one other pathogenic bacterium was shown to bind C1-inh. *Escherichia coli* strain 0157:H7 can bind C1-inh to its surface via the metalloprotease StcE, to evade the complement system [41, 42].

The host complement regulator C4b-binding protein (C4BP) also inhibits complement activation via the CP and LP. C4BP is a spider-like molecule of 570 kDa, composed of seven identical α-chains and one β-chain held together by di-sulfide bridges. Both chains are composed of complement control protein domains (CCPs). C4BP binds to C4b, thereby dissociating the CP/LP C3 convertase C4b2a and it acts as a cofactor for the plasma protease factor I in the proteolytic degradation of C4b [10]. *B. pertussis* binds C4BP via its surface protein filamentous hemagglutinin (FHA) [43] (Fig. 3c). Although C4BP binding to *B. pertussis* occurs under physiological conditions and C4BP retains its complement regulatory activity when surface-bound [44], protection from complement-mediated lysis has not been proven. *B. pertussis* mutants that do not express FHA have a similar sensitivity toward complement compared to the wild-type strain [45]. Studies using C4BP mutants show that *B. pertussis* binds C4BP at the CCP1-2 domain interface of the α-chain. Amino acids R64 and R66 of C4BP are the major players in the binding site. Moreover, studies with mAb directed towards the C4BP α-chain indicate that *B. pertussis* binds C4BP at a site similar to the C4b-binding site [44]. Although strongly reduced, *fha* mutants were still able to bind C4BP. *bvg* mutants failed to bind C4BP [43] indicating that besides FHA, one or more BvgAS-regulated proteins contribute to the binding of C4BP (Fig. 2b). Binding of C4BP to bacterial pathogens is a common phenomenon. *Neisseria meningitidis* binds C4BP via its type IV Pili [46], group A streptococcus binds C4BP via the M-protein family member Sir and Arp [47, 48] and also *E. coli* strain K1 binds C4BP via outer membrane protein A [49].

Finally, *B. pertussis* and *B. parapertussis* are both capable of binding host-derived negative complement regulator fH family proteins including factor H-like (FHL)-1 and factor H-related (FHR)-1. Binding of fH family proteins by pathogens contributes to their survival in human serum [50] (Fig. 3d). FH, the main negative regulator of the AP, consists of 20 short consensus repeat (SCR) domains and recent studies show that *B. pertussis* binds fH via SCR20 and SCR5-7 [51, 50]. FH binding by other pathogenic bacteria such as *Pseudomonas aeruginosa*, *Haemophilus influenza*, and *Streptococcus pneumoniae* occurs via SCR19-20 through a common site in SCR20 named the “common microbial binding site.” This binding site allows the formation of a tripartite microbial protein: fH:C3b complex which enhances fH-mediated inactivation of C3b. A similar mechanism of complement evasion has not yet been shown for *B. pertussis* [51] since the fH binding protein(s) of *B. pertussis* remains unidentified. Ptx could be involved in fH binding since an isogenic strain of Tohama I, lacking Ptx, is more sensitive to serum killing via the AP than the parental strain [50]. Taken together, *B. pertussis* produces various complement evasion molecules to successfully colonize and persist in the human host. We hypothesize that *B. pertussis* expresses more, yet unidentified, molecules for regulating complement activation since other well studied bacteria have proven to express at least a dozen.

## Complement evasion molecules and vaccine development

The recent increase in pertussis cases worldwide has made it clear that further research on the pathogenesis and immunity of *B. pertussis* is required for the development of a new generation of vaccines. Besides humans, no other reservoirs for *B. pertussis* have been described. Therefore, strain adaptation is driven by the human immune system only and waning immunity may accelerate this process by allowing a high circulation rate of the pathogen [52]. Next-generation *B. pertussis* vaccines should include antigen preparations that induce long-lasting immunity and strengthens the innate immune system. Strengthening innate immune responses will result in elimination of *B. pertussis* immediately after exposure to this bacterium, preventing transmission and thereby, opportunities for strain adaptation.

New-generation vaccines against a number of pathogens are currently converging on the use of complement evasion molecules as vaccine targets. The best example is factor H binding protein (fHbp) of *N. meningitidis*, which is the leading antigen in vaccine development against *N. meningitidis* serogroup B [53]. fHbp binds to fH providing an important mechanism for immune evasion by inhibition of the complement system [54, 55]. Next to the vaccine against *N. meningitidis*, other vaccines containing complement evasion molecules are also investigated [56, 57]. BibA is a virulent factor from group B *Streptococcus* that is able to bind to C4BP and promotes adhesion of group B *streptococcus* to human epithelial cells [58]. Recent studies also indicate pneumococcal proteins PspA and PspC as potential vaccine candidates. PspA and PspC are important virulence factors expressed by almost all pneumococcal strains. Both proteins are known as complement evasion molecules. PspA interferes with complement deposition on the bacterial surface and PspC binds to fH [59, 60].

The aP vaccine for *B. pertussis* consists of Ptx, Fim, FHA, and Prn (depending on which country). As described above in detail, FHA is a known C4BP-binding molecule [43, 44]. Recent studies show that vaccination with Vag8, the C1-inh binding protein of *B. pertussis*, results in a protective immune response against *B. pertussis* in mice [52]. All these data together underline the promising perspective of using complement modulating proteins in new vaccines.

Studies to identify additional molecules involved in complement evasion by *B. pertussis* are needed to develop new and improved vaccines against *B. pertussis*. Vaccines containing *B. pertussis* complement evasion molecules will prevent complement escape and complement downregulation. Effective activation of the complement system is important since the function of the complement system goes beyond protecting the host from infection by immediate elimination of pathogens; it is also involved in modulation of adaptive immune responses [14]. Altogether, it is of crucial importance to unravel the complement evasion strategies of *B. pertussis* in order to improve the existing vaccines against pertussis.

## Concluding remarks

Despite high vaccination coverage, reported cases of pertussis are rising which clearly indicates the urgency for a more effective vaccine [9]. The role of the complement system in protection against *B. pertussis* has only recently become apparent [25, 61], and research on complement evasion by *B. pertussis* is limited. We propose that improved pertussis vaccines should contain antigen preparations that, in addition to inducing long-lasting immunity, can prevent suppression of the innate immune response by *B. pertussis*. One of the possibilities is inclusion of complement evasion molecules in current aP vaccines. Neutralizing complement evasion molecules would allow more efficient activation of the complement system upon exposure to *B. pertussis*, which will result in faster eradication of the bacteria and potentially leads to a better activation of the adaptive immunity. In conclusion, complement evasion molecules are undoubtedly promising vaccine candidates.
